# Fast-Flu: RT-RPA-CRISPR/Cas12a assisted one-step platform for rapid influenza B virus detection

**DOI:** 10.1128/spectrum.00365-25

**Published:** 2025-04-25

**Authors:** Dayong Xu, Qianlin Wu, Fo Yang, Qi Zhang, Qiuyang Jiang, Xiaotong Zeng, Yushuo Zhang, Tingyao Lv, Jin Wang, Feng Li

**Affiliations:** 1 Anhui Province Key Laboratory of Pollutant Sensitive Materials and Environmental Remediation, Huaibei Normal University 58286, Huaibei, Anhui, China; 2 School of Life Sciences, Huaibei Normal University 58286, Huaibei, Anhui, China; 3 Huaibei People’s Hospital, Huaibei, Anhui, China; 4 Department of Clinical Laboratory, Shenzhen Institute of Translational Medicine, The First Affiliated Hospital of Shenzhen University, Shenzhen Second People’s Hospital 629318, Shenzhen, China; 5 Tolo Biotechnology Co., Ltd, Wuxi, Jiangsu, China; Children's National Hospital, George Washington University, Washington, DC, USA

**Keywords:** Flu B detection, RT-RPA, CRISPR/Cas12a, one-step, Fast-Flu

## Abstract

Influenza B virus (Flu B) is a prevalent respiratory pathogen responsible for seasonal influenza epidemics. Despite its clinical significance, there remains a lack of rapid and accurate diagnostic methods for Flu B detection. In this study, we developed a novel Flu B detection system, named Fast-Flu, by integrating reverse transcription recombinase polymerase amplification (RT-RPA) with the clustered regularly interspaced short palindromic repeats (CRISPR)-associated protein (Cas) system (CRISPR/Cas). Through optimization of reaction temperature and adjustment of Cas12a concentrations, we successfully balanced RPA amplification and CRISPR/Cas12a trans-cleavage activity, enabling the establishment of a one-step detection system. The one-step Fast-Flu system demonstrated the ability to specifically identify Flu B within 45 min, with a limit of detection of 58 copies per test. It eliminates the need for uncapping operations and minimizes the risk of cross-contamination, without cross-reactivity with other pathogens. When evaluated using 101 clinical throat swab samples, the one-step Fast-Flu system achieved a sensitivity of 56.25% and a specificity of 100% compared to the PCR-based method, with an overall concordance rate of 93.06% (94/101). The development of this one-step RT-RPA-CRISPR/Cas12a system represents a significant advancement in the rapid, convenient, and accurate detection of Flu B, highlighting its potential for clinical diagnosis. Furthermore, with future technical improvements to enhance sensitivity, this one-step RT-RPA-CRISPR assay holds promise as a versatile tool for the rapid nucleic acid detection of other RNA viruses.

**IMPORTANCE:**

Influenza B virus (Flu B) is a significant global health concern, and rapid, accurate pathogen diagnosis is crucial for effective influenza prevention and control. The integration of isothermal amplification methods with the clustered regularly interspaced short palindromic repeats (CRISPR)/CRISPR-associated protein (Cas) system has achieved high sensitivity and specificity for nucleic acid detection. Although CRISPR/Cas-based systems have been developed for influenza detection, existing platforms require the transfer of amplified products into the CRISPR/Cas12a detection system through uncapping operations, which increases the risk of cross-contamination. In this study, we developed a one-step reverse transcription recombinase polymerase amplification-CRISPR/Cas12a Flu B detection method using a one-pot detection system. By optimizing the reaction temperature and Cas12a concentration, we achieved a streamlined and contamination-free workflow. This innovative approach not only improves Flu B detection but also serves as a valuable reference for constructing CRISPR/Cas systems for the detection of other pathogens and targets, paving the way for broader applications in molecular diagnostics.

## INTRODUCTION

Influenza B virus (Flu B) is a highly contagious respiratory pathogen that causes acute febrile illness, posing a significant global health burden (1). Annually, billions of infections and deaths are attributed to Flu B, with its prevalence rapidly increasing (2). Flu B typically peaks during winter seasons and can lead to severe, life-threatening complications such as encephalitis, bacterial pneumonia, sinus infections, myositis, and Reye’s syndrome (3). Although vaccinations and antiviral drugs have contributed to reducing the incidence of Flu B infections, their effectiveness remains limited due to the virus’s ability to evade detection through antigenic variation. Consequently, there is an urgent need for a rapid and accurate diagnostic method for Flu B to mitigate its impact and optimize clinical treatment strategies.

Flu B is an RNA virus belonging to the *Orthomyxoviridae* family (4). Conventional diagnostic methods for Flu B include virus isolation, antigen testing, and serological assays (5–7). However, these methods are time-consuming and unsuitable for rapid diagnosis. In recent years, molecular diagnostic tests, particularly PCR-based methods, have become widely used for detecting respiratory viruses, including Flu B (8). Despite their utility, PCR-based methods are restricted by their reliance on expensive equipment, skilled technicians, and lengthy reaction times. In contrast, the isothermal amplification techniques offer a promising alternative for Flu B detection, such as loop-mediated isothermal amplification assay (LAMP) (9, 10) and recombinase polymerase amplification (RPA), which operate at a constant temperature without the need for specialized thermal cycles and consumed reagents (11, 12). However, the generation of non-specific amplification products often compromises the specificity of these methods (13, 14).

The clustered regularly interspaced short palindromic repeats (CRISPR)-associated protein system (Cas) (CRISPR/Cas), conventionally used for genome editing, has recently been adapted for nucleic acid detection (15). For molecular diagnostics, the CRISPR/Cas system is proven with high specificity and sensitivity (16–20). Upon a targeting sequence binding with its antisense CRISPR RNA (crRNA), CRISPR/Cas12a starts its cis-cleavage activity on the target sequence, followed by its trans-cleavage activity on the single-stranded DNA (ssDNA) reporter probe labeled with fluorescein, which results in the release of the fluorescence signal (21). The CRISPR/Cas system is able to discriminate even single-base mismatches, which guarantees exceptional specificity. Consequently, RPA is presently combined with the CRISPR/Cas system to serve as a powerful approach for nucleic acid detection, ensuring both high sensitivity and specificity. While CRISPR-based systems have been developed for influenza typing and detection (22–25), these platforms typically require transferring amplified products into the CRISPR/Cas12a detection system through uncapping operation, which increases the risk of cross-contamination.

A one-step reverse transcription (RT)-RPA-CRISPR/Cas12a Flu B detection system integrates the reagents for RT-RPA and CRISPR/Cas12a assays into a single reaction, significantly streamlining the operational process. Nevertheless, the dual cis-cleavage and trans-cleavage activities of Cas12a present a challenge, as the cis-cleavage on the template and amplification products can substantially reduce detection sensitivity. In this study, we seek to address this challenge by optimizing the reaction temperature and the concentration of Cas12a to minimize cis-cleavage effects on the template and amplification products, thereby enhancing detection sensitivity. This study aims not only to improve the performance of the Flu B detection system but also serves as a valuable reference for developing the CRISPR/Cas system for the detection of other pathogens and targets.

## MATERIALS AND METHODS

### RPA primer design and selection for Flu B

The nucleic acid sequence of Flu B was downloaded from the NCBI database (https://www.ncbi.nlm.nih.gov/), and the RPA primers were designed according to the specific conserved region. The sequences of primers were checked in the NCBI Basic Local Alignment Search Tool database to ensure there is no cross-reactivity with other viruses/microbes. Three forward (F1 to F3) and reverse (R1 to R3) primers were designed and synthesized by Sangon Biotech (Shanghai, China). Their sequences, as well as their positions and entropy values, are listed in Table 1. Moreover, the RNA extracted from the purchased commercial Flu B pseudovirus product (DP315, Beyotime, China) acted as the template. Then, the forward and reverse primers were pair-wise combined and used for RPA amplification conducted with a commercial kit (KS102), with 1,000 copies per test of RNA template incubated at 38°C for 30 min. For each primer pair, the information on the size of the amplification product and the melting temperature (Tm) is listed in Table 2. The product quality was examined via agarose gel electrophoresis to select the best primer combinations.

**TABLE 1:** The sequences, positions, and entropy values of RPA primers^*a*^

Primers	Sequences	Positions	Entropy values (△S: cal/(°K * mol))
F1	5´-TTGCYACTGATGATCTTACAGTGGAGGATGAA-3´	653–684	643–656.8
F2	5´-TGTTGCYACTGATGATCTTACAGTGGAGGATG-3´	651–682	644.1–657.9
F3	5´-CTTGTT GCYACTGATGATCTTACAGTGGAGGA-3´	648–680	646.2–660
R1	5´-TCTGGTGATAATCGGT GCTCTTGACCAAATTG-3´	784–815	683.3
R2	5´-TGATAATCGGTGCTCTTGACCAAA TTG GGATA-3´	779–810	682.4
R3	5´-TCGGTGCTCTTGACCAAATTGGGATAAGACTC-3´	773–804	694.4

^*a*^ F1 to F3, forward primers; R1 to R3, reverse primers.

**TABLE 2:** The information on the size of the amplification product and the Tm of each primer pair

Primer pairs	Product length (bp)	Tm range (°C)
F1R1	163	61–62
F1R2	158	61–62
F1R3	152	61–63
F2R1	165	62–63
F2R2	160	61–63
F2R3	154	62–63
F3R1	167	62–63
F3R2	162	61–63
F3R3	156	62–63

### Design and selection of crRNA for one-pot RT-RPA-CRISPR/Cas12a systems

According to the sequences of the selected primers, six Cas12a crRNAs (crRNA1 to crRNA6) were designed for selection in one-pot and one-step Flu B detection systems. The sequences of crRNAs were shown as follows, and the target sequences were underlined: crRNA1: 5′-UAAUUUCUACUAAGUGUAG AUAAAGCCAAUUCGAGCAGCUG-3′, crRNA2: 5′-UAAUUCUACUAAGUG UAGAUGAGCAGCUGAAACUGCGGUG-3′, crRNA3: 5′-UAAUUUCUACUA AGUGUAGAUCACCGCAGUUUCAGCUGCUC-3′, crRNA4: 5′-AAUUUCUACU AAGUGUAGAUCAGCUGCUCGAAUUGGCUUU-3′, crRNA5: 5′-UAAUUUC UACUAAGUGUAGAUCAGCUGCUCGAAUUGGCUUU-3′, crRNA6: 5′-UAAUUUCUACUAAGUGUAGAUAGCUGCUCGAAUUGGCUUUG-3′. All the crRNAs were synthesized by using the commercial Cas12a High Yield crRNA Synthesis and Purification Kit (31903-01, TOLOBIO, China) according to the instruction given by the manufacturer.

### Condition optimization of the one-pot RT-RPA-CRISPR/Cas12a system

The one-pot Flu B detection system includes two parts, RT-RPA amplification and CRISPR/Cas12a detection. The total volume of the RT-RPA assay was 24 µL, including 1.05 µL of FluB-F (10 µM), 1.05 µL of FluB-R (10 µM), 5 µL of RNA Template (100 copies per microliter), 1 µL of activator, and 15.9 µL of nuclease-free water. In the one-pot Flu B detection system, the RT-RPA reaction was performed with 500 copies per test of RNA (derived from the Flu B pseudovirus) at 38°C on a QuantStudio 3 real-time fluorescence quantitative PCR system (QuantStudio 3, Thermo Fisher, USA) for 30 min.

The CRISPR/Cas12a detection system contains 3.0 µL of HOLMES Buffer (10×), 0.75 µL of Lb5Cas12a (10 µM, 32110, ToLo Biotech, China), 0.75 µL of crRNA (10 µM), and 1.5 µL of ssDNA reporter (10 µM, 31101, ToLo Biotech, China). CRISPR/Cas12a system was incubated for 10 min. Different settings in working temperature and concentration for Cas12a, concentration of crRNA6, and ssDNA probe reporters are demonstrated in Table 3. The CRISPR/Cas12a system was performed in a reaction volume of 10 µL and maintained for 10 min at various gradients of temperature. Then, the fluorescence intensity was collected every 30 s.

**TABLE 3:** Condition optimization of the one-pot RT-RPA-CRISPR/Cas12a system

Items	Conditions
Working temperature for Cas12a (°C)	38, 40, 42, 44, 46, 48, 50, 52, 54
Concentration of Cas12a (nM)	62.5, 125, 250, 500
Concentration of crRNA6 (nM)	250, 375, 500, 625
ssDNA probe reporters	
P8A	6´FAM-AAAAAAAA-BHQ1
P8C	6´FAM-CCCCCCCC-BHQ1
P8T	6´FAM-TTTTTTTT-BHQ1
P8G	6´FAM-GGGGGGGG-BHQ1

### Condition optimization of one-step RT-RPA-CRISPR/Cas12a system

The total volume of one-step Flu B detection system was 25 µL, including 1.05 µL of FluB-F (10 µM), 1.05 µL of FluB-R (10 µM), 5 µL of RNA template (500 copies per microliter), 1 µL of RNA template, and 1 µL of activator, 1.25 µL of Lb5Cas12a (10 µM), 2.5 µL of crRNA (10 µM), 0.5 µL of reporter (10 µM), and 12.65 µL of nuclease-free water.

The working temperature and concentration for Cas12a, concentration of crRNA6, and ssDNA probe reporters were set at different conditions, which are detailed in Table 4. The reaction was performed for 45 min at different gradients of temperature, and the fluorescence signal was collected every 1 min.

**TABLE 4:** Condition optimization of the one-step RT-RPA-CRISPR/Cas12a system

Items	Conditions
Working temperature for Cas12a (°C)	36, 38, 40, 42, 44
Concentration of Cas12a (nM)	62.5, 125, 250, 500
Concentration of crRNA6 (nM)	125, 188, 250, 313
ssDNA probe reporters	
P8A	6´FAM-AAAAAAAA-BHQ1
P8C	6´FAM-CCCCCCCC-BHQ1
P8T	6´FAM-TTTTTTTT-BHQ1
P8G	6´FAM-GGGGGGGG-BHQ1

### Sensitivity analysis of the one-step RT-RPA-CRISPR/Cas12a for Flu B detection

To determine the limit of detection (LOD) of one-pot and one-step Flu B detection method, the recombinant plasmid of Flu B was constructed by using a Flu B pseudovirus sample, which contains a segment of Flu B (GenBank: KC844196.1). The recombinant plasmid was serially diluted into 200 copies per test, 100 copies per test, 50 copies per test, and 25 copies per test. Ten replicates were required for each reaction at each dose. The LOD of the present method was predicted by using the sigmoid function according to the positive results of each dose.

### Specific detection of the one-step RT-RPA-CRISPR/Cas12a for Flu B

To determine the specificity, a family of six interfering samples were collected for detection, including Group A *Streptococcus* (GAS), *Acinetobacter baumannii* (AB), human parainfluenza virus (HPIV), *Mycoplasma pneumoniae* (MP), *Klebsiella pneumoniae* (KP), and H1N1 avian influenza (H1N). The Flu B pseudoviral nucleic acid served as the positive control (PC), while the nuclease-free water was used as the no template control (NTC). All the samples were detected by using the one-step RT-RPA CRISPR/Cas12a detection system, with reaction performed in triplicate.

### Validation of the RT-RPA-CRISPR/Cas12a system in human clinical samples

To verify the Flu B detection performance of the RT-RPA-CRISPR/Cas12a system, a total of 101 throat swab samples were provided by the institution of Huaibei People’s Hospital. They were obtained from 101 patients including 60 males and 41 females, aged from 5 days to 91 years old.

RNA samples were extracted from human throat swab samples by using the commercial kit (DP315, Beyotime, China) according to the instruction provided by the manufacturer and used as the template in RT-RPA reaction for target sequence amplification. All the samples were simultaneously detected by using the present one-pot and one-step RT-RPA-CRISPR/Cas12a methods, respectively. The result of the PCR-based method served as the gold standard. Then, the consistency of one-pot or one-step with PCR-based method was analyzed according to the detection results.

### Data processing and statistical analysis

The detection results were indicated by relative fluorescence fold of individual samples. Each sample was detected for at least three biological replicates, and the data were represented by mean ± SD. The GraphPad Prism 10 software (GraphPad Software Inc., CA, USA) was applied for statistical analysis. Statistical differences were evaluated via the Student’s *t*-test. The LOD was predicted by using the sigmoid function. A value of *P* < 0.05 was considered statistically significant.

## RESULTS

### The workflow of one-pot and one-step RT-RPA-CRISPR/Cas12a systems for Flu B detection

**Fig 1:**
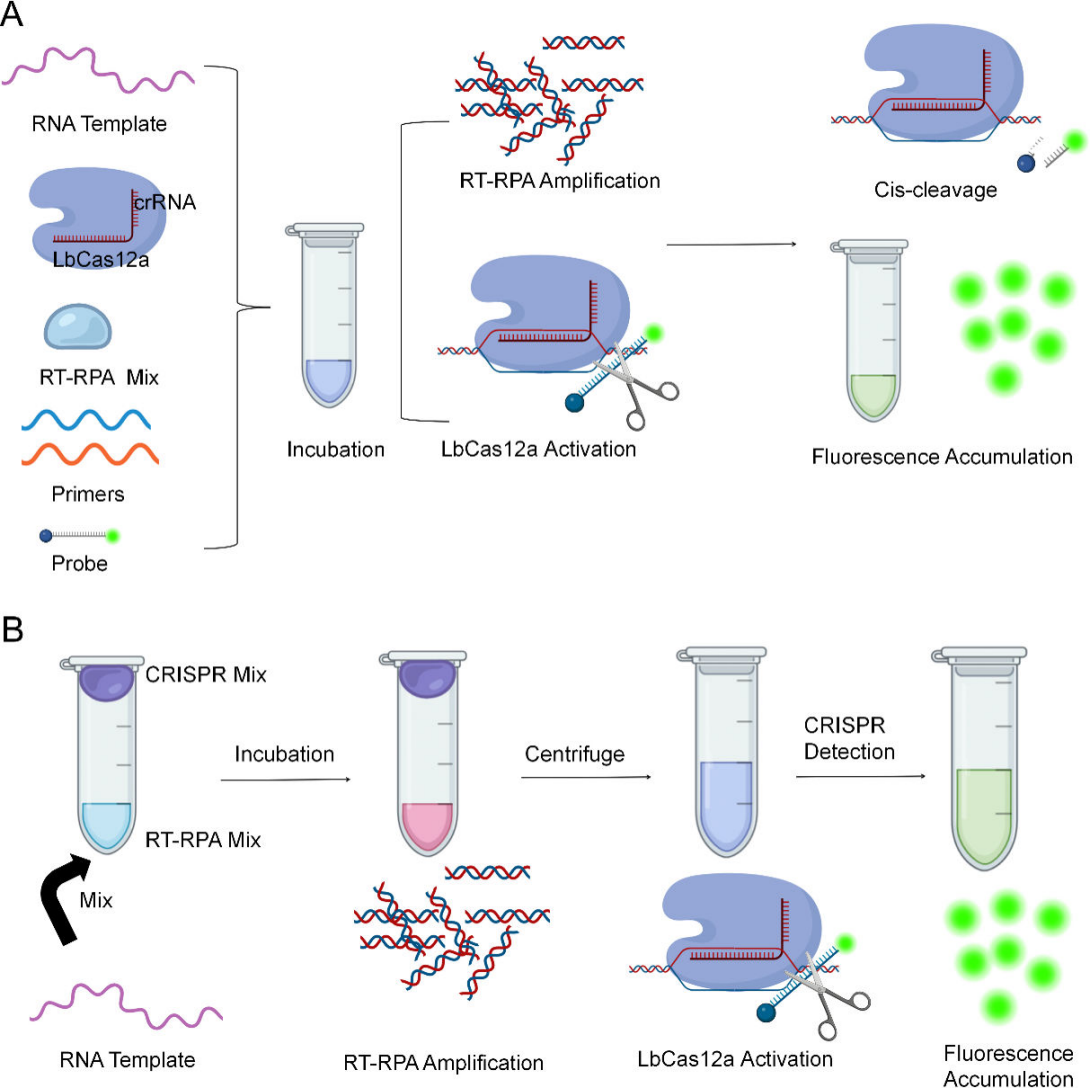
The workflow of one-pot and one-step RT-RPA-CRISPR/Cas12a system for Flu B detection. (A) For one-pot Flu B detection system, the RT-RPA assay was prepared at the bottom of the tube, and the CRISPR/Cas12a detection system was set in the cap of the tube. After amplification, the CRISPR/Cas12a reaction was added into the RT-RPA system via a short spin. The Cas12a can be activated in the presence of the target sequence and trans-cleaved FAM-labeled ssDNA probe to release the fluorescence signal. (B) For the one-step Flu B detection system, both the RT-RPA reaction and CRISPR/Cas12a detection system were mixed at the bottom of the tube. Then the reaction was performed at a constant temperature on a fluorescence signal detector. The Flu B-positive samples will induce a remarkable fluorescence signal, while no significant signal can be observed for Flu B-negative samples.

The schema of the present workflow for one-pot RT-RPA-CRISPR/Cas12a Flu B detection systems was illustrated in column A of Fig. 1. Briefly, both the RT-RPA assay and CRISPR/Cas12a reaction were prepared in one tube. The RT-RPA reaction proceeded at the tube bottom, while the CRISPR/Cas12a detection system was prepared in the tube cap. After the target sequence of Flu B was amplified by the RT-RPA reaction, the CRISPR/Cas12a detection system was centrifuged into the RT-RPA reaction system via a short spin. With the aid of a FAM-labeled ssDNA probe, the fluorescence signal would be released and visualized on a fluorescence signal detector. A remarkable fluorescence signal could emerge when detecting a positive Flu B sample, while no significant signal could be detected in negative Flu B samples. This one-pot detection system can be finished within 40 min, including 30 min of RT-RPA and 10 min of CRISPR/Cas12a detection.

In the one-step RT-RPA-CRISPR/Cas12a system, the reagents for RT-RPA and CRISPR/Cas12a assays were integrated into a single reaction, and the reaction was finished within 45 min and analyzed on a fluorescence signal detector. The schema of the present workflow for one-step RT-RPA-CRISPR/Cas12a Flu B detection systems was demonstrated in column B of Fig. 1.

### Selection of RT-RPA primer for Flu B amplification

**Fig 2:**
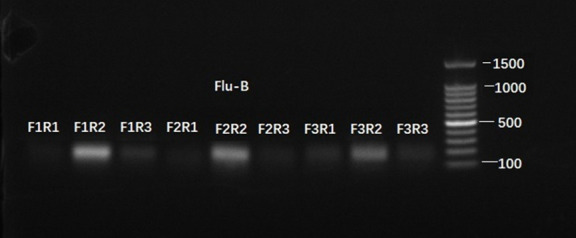
The primer selection for Flu B amplification in RT-RPA reaction. According to the conserved region of Flu B, three forward primers (F1 to F3) were designed and combined with three reverse primers (R1 to R3), respectively. RNA extracted from the Flu B pseudovirus acted as the detection template (1,000 copies per test). RPA amplification was performed at 38°C according to the recommendation by the kit. Their amplification products were examined by performing a 2% agarose gel electrophoresis.

Based on the specific conserved region of Flu B nucleic acid sequence, three forward (F1 to F3) and three reverse (R1 to R3) primers were designed for the RT-RPA assay. They were pair-wise combined into nine primer combinations and employed in the RT-RPA assay for Flu B amplification. To select the optimum primer combination, their amplification products were subjected to quality testing by performing agarose gel electrophoresis. According to the product yield, the F1/R2 was settled as the optimal primer combination (Fig. 2).

### Selection of crRNA for one-pot RT-RPA-CRISPR/Cas12a system

**Fig 3:**
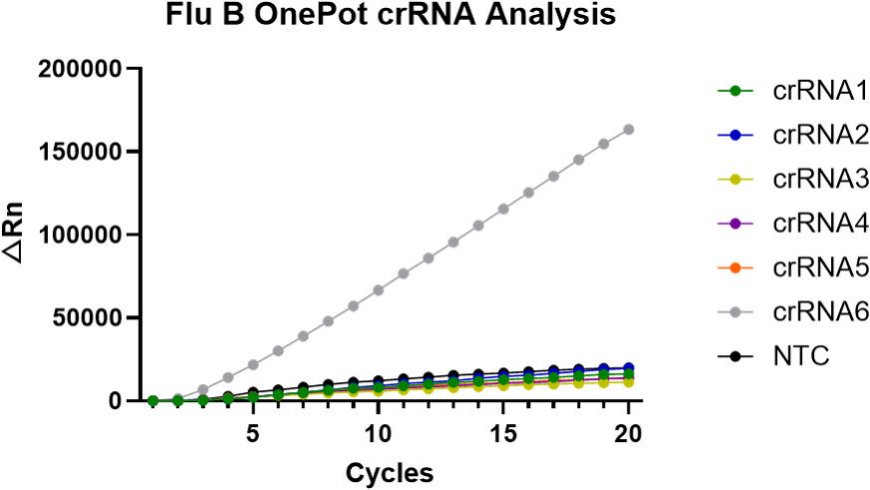
The selection of crRNA for one-pot RT-RPA-CRISPR/Cas12a system. Based on the sequences of the target gene, a total of six crRNAs were designed (crRNA1 to crRNA 6), and they were evaluated and selected for one-pot Flu B detection system. The RNA template (500 copies per test) derived from the Flu B pseudovirus was amplified through the RPA assay in 10 µL of reaction system by incubating at 38°C for 30 min. The CRISPR/Cas12a system was performed in a reaction volume of 10 µL, by maintaining at 48°C for 10 min.

According to the amplified nucleic acid sequence of Flu B, six Cas12a crRNAs (crRNA1 to crRNA 6) were synthesized for selection. In conducting the one-pot Flu B detection system, we observed that the detection efficiency reached the highest when using crRNA 6 (Fig. 3). Consequently, crRNA6 was selected for the one-pot Flu B detection system.

### Condition optimization of the one-pot RT-RPA-CRISPR/Cas12a system

**Fig 4:**
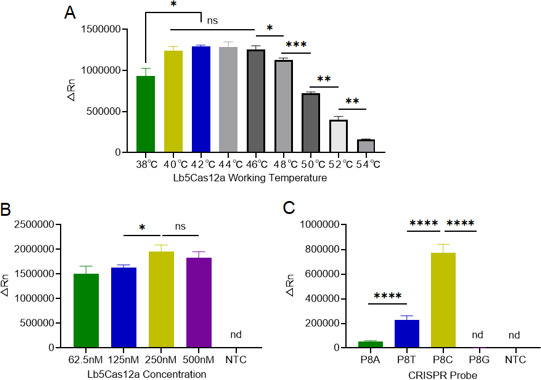
The condition optimization for one-pot Flu B detection system. (A) The optimal working temperature of Lb5Cas12a was selected, with 500 copies per test RNA template used, RT-RPA reaction performed at 38°C for 30 min, CRISPR/Cas12a maintained at 48°C for 10 min (*n* = 3 replicates, Student’s *t*-test; *P < 0.05, **P < 0.01, ***P < 0.001; bars represent mean ± SD; ns, no significance). (B) The best concentration of Lb5Cas12a was analyzed, with 500 copies per test RNA template used, RT-RPA reaction performed at 38°C for 30 min, CRISPR/Cas12a maintained at 48°C for 10 min (*n* = 3 replicates, Student’s *t*-test; *P < 0.05; bars represent mean ± SD; ns, no significance). (C) The performance of different probes was examined, with 500 copies per test RNA template used, RT-RPA reaction performed at 38°C for 30 min, CRISPR/Cas12a maintained at 48°C for 10 min (*n* = 3 replicates, Student’s *t*-test; **P < 0.01, ***P < 0.001, ****P < 0.0001; bars represent mean ± SD; nd, not detected).

Based on our previous result, the temperature of the RT-RPA reaction was set at 38°C. Next, the working temperature of Cas12a was selected from 38°C to 54°C by 2°C increments. As shown in Fig. 4A, it revealed the highest fluorescence intensity when the working temperature of Cas12a was at 42°C, which was ascertained as the optimal temperature.

In the one-pot Flu B detection system, the concentration of Cas12a was diluted into 62.5 nM, 125 nM, 250 nM, and 500 nM, respectively, and the result suggested that the best performance appeared at the concentration of 250 nM for Cas12a (Fig. 4B).

After that, the ssDNA FAM-labeled probe of P8C showed better performance than the other three probes, and then P8C was selected as the probe used in the one-pot Flu B detection reaction (Fig. 4C).

### Condition optimization of one-step RT-RPA-CRISPR/Cas12a system

**Fig 5:**
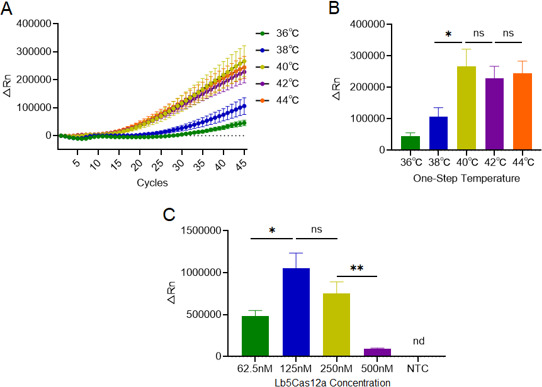
The performance test of the one-step Flu B detection system. A total of 400 copies per test of RNA derived from the Flu B pseudovirus was used as template, and the reaction was performed in 10 µL of system. (A) The fluorescence intensity detected in systems at different working temperatures of Lb5Cas12a. (B) The optimal working temperature of Lb5Cas12a was selected (*n* = 3 replicates, Student’s *t*-test; *P < 0.05; bars represent mean ± SD; ns, no significance). (C) The best concentration of Lb5Cas12a was analyzed (*n* = 3 replicates, Student’s *t*-test; *P < 0.05, **P < 0.01; bars represent mean ± SD; ns, no significance; nd, not detected).

According to the above conditions, we further optimize the reaction condition of the one-step Flu B detection system, including the optimal reaction temperature and concentration of Cas12a. Firstly, the reaction temperature of Cas12a was set ranging from 36°C to 44°C by 2°C increments. Clearly, the fluorescence intensity at 40°C was significantly higher than at 38°C, while no significant difference was found between 40°C and 42°C. As a consequence, the temperature for the one-step Flu B detection system was intended at 40°C (Fig. 5A and B).

The concentration of Lb5Cas12a was diluted into 62.5 nM, 125 nM, 250 nM, and 500 nM, respectively, and the data suggested that the fluorescence intensity reached the highest with a concentration of 125 nM (Fig. 5C). When the concentration of Lb5Cas12a exceeded 125 nM, the fluorescence intensity was decreased, implying the diminished trans-cleavage reaction and enhanced cis-cleavage reaction.

### Sensitivity analysis on the one-pot and one-step RT-RPA-CRISPR/Cas12a system for Flu B detection

**Fig 6:**
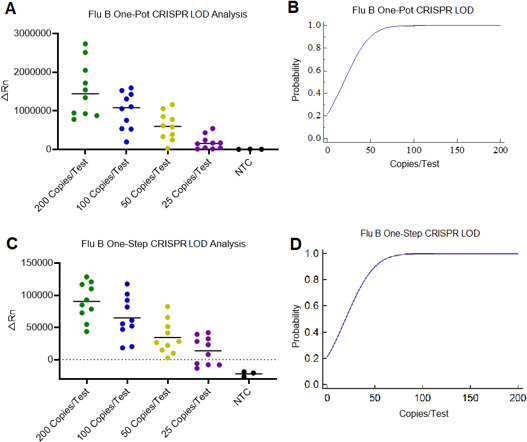
Condition optimization of the one-pot and one-step Flu B detection system. (A) The fluorescence intensities detected in template plasmid at different doses, when performing the one-pot Flu B detection system (*n* = 10 replicates). (B) The LOD of the one-pot Flu B detection system was predicted by using the sigmoid function. (C) The fluorescence intensities detected in template plasmid at different doses, when performing the one-step Flu B detection system (*n* = 10 replicates). (D) The LOD of the one-step Flu B detection system was predicted by using the sigmoid function.

To determine the sensitivity of the one-pot and one-step RT-RPA-CRISPR/Cas12a Flu B detection method, the recombinant plasmid was diluted into 200 copies per test, 100 copies per test, 50 copies per test, and 25 copies per test, respectively. Ten replicates were performed at each gradient. In the one-pot RT-RPA-CRISPR/Cas12a detection, 10 positive results were identified in the gradients of 200 copies per test and 100 copies per test, while 9 positive results in 50 copies per test and 6 positive results in 25 copies per test were determined (Fig. 6A). The detection rates were 100%, 100%, 90%, and 60%, respectively. Then, the sigmoid plot function elucidated an LOD of 58 copies per test at the probability of 95%, by using the one-step RT-RPA-CRISPR/Cas12a system for Flu B detection (Fig. 6B). In the one-step RT-RPA-CRISPR/Cas12a detection, the same way was used to calculate the LOD. In this test, the ΔRn = 0 was used as the threshold value. The one-step detection has the same detection rates and LOD value as the results of one-pot detection (Fig. 6C and D).

### Specific detection of Flu B by the one-step RT-RPA-CRISPR/Cas12a system

**Fig 7:**
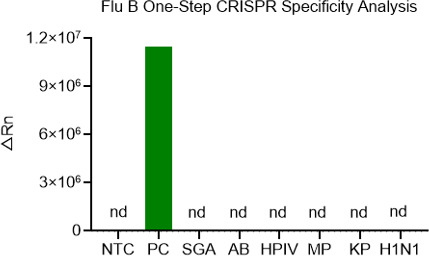
The specificity of the RT-RPA-CRISPR/Cas12a system for Flu B detection. Six interfering nucleic acid samples were used to analyze the specificity of the one-step RT-RPA CRISPR/Cas12a system for Flu B detection, with the Flu B pseudoviral nucleic acid serving as the positive control, while the nuclease-free water was used as the NTC.

In order to verify the specific detection of the one-step system for Flu B, a total of six interfering nucleic acid samples were collected for detection, including GAS, AB, HPIV, MP, KP, and H1N. The Flu B pseudoviral nucleic acid served as the PC, while the nuclease-free water was used as the NTC. As illustrated in Fig. 7, only the PC samples produced a significant fluorescence intensity, while no significant signal was observed in the interfering samples. These results demonstrated that the one-step Flu B detection system was highly specific for Flu B detection, without any cross-reaction with other nucleic acid samples.

### Performance validation of the one-step RT-RPA-CRISPR/Cas12a systems for Flu B detection in clinical samples

To examine the performance of the present method in the real-world clinical samples, a total of 101 throat swab samples were collected for validation. All the samples were simultaneously detected by using one-pot and one-step RT-RPA-CRISPR/Cas12a Flu B detection method, respectively. Meanwhile, the results of the commercial PCR-based Flu B detection kit were taken as the gold standard. As shown in Table 5, there were 16 positive and 85 negative results identified by using the PCR-based method. When employing the one-pot Flu B detection system, the positive and negative samples were 14 and 87, respectively, with a sensitivity of 81.25% and a specificity of 98.82%. Among these two methods, a sum of 97 samples was in agreement, and the consistency between PCR-based and one-pot methods was 96.03% (97/101).

**TABLE 5:** Comparison between the performance of the one-pot RPA-CRISPR/Cas12a method and qPCR for Flu B detection

One-pot	qPCR		Sum	Sensitivity	Specificity	Consistency
	No. positive	No. negative				
Positive	13	1	14	81.25%	98.82%	96.03%
Negative	3	84	87			
Total	16	85	101			

A total of 9 positive and 92 negative samples were identified by using the one-step Flu B detection system, with a sensitivity of 56.25% and a specificity of 100% (Table 6). Among them, the result of 94 samples was agreed with the PCR-based method, including 9 positive and 85 negative results, respectively. The consistency of these two methods was 93.06% (94/101) in all 101 samples.

**TABLE 6:** Comparison between the performance of the one-step RPA-CRISPR/Cas12a method and qPCR for FluB detection

One-step	qPCR		Sum	Sensitivity	Specificity	Consistency
	No. positive	No. negative				
Positive	9	0	9	56.25%	100%	93.06%
Negative	7	85	92			
Total	16	85	101			

## DISCUSSION

In this study, we developed a one-step Flu B detection method by integrating RT-RPA assay and CRISPR/Cas12a technology into a single-tube reaction. This system demonstrated the ability to specifically identify Flu B within 45 min, with an LOD of 58 copies per test. To evaluate its performance, we compared the one-step Flu B detection system with a PCR-based method using 101 clinical throat swab samples. The results revealed a sensitivity of 56.25%, a specificity of 100%, and an overall consistency of 93.06% between the two methods. In summary, this system enables rapid and accurate detection of Flu B, contributing to early diagnosis and precise treatment of influenza B.

So far, the RT-RPA-based platform was employed in screening of influenza viruses (Flu A and Flu B) and severe acute respiratory syndrome coronavirus 2 (SARS-CoV-2) within 1 hour, reaching a sensitivity as low as 10 copies of viral RNA (26, 27). Nevertheless, the false-positive results caused by the non-specific amplification products restricted the application of isothermal amplification techniques (28–30). An RPA-CRISPR/Cas12a-based Flu B detection method achieved a sensitivity of 1 plaque-forming unit per reaction without cross-reactivity (23, 31). However, these platforms typically involve two separated steps—target sequence RPA and CRISPR/Cas12a detection—requiring uncapping operation that increases the risk of aerosol contamination. Additionally, reagent preparation and centrifugation steps add complexity, hindering clinical translation. To overcome these challenges, we developed a one-step RT-RPA-CRISPR/Cas12a system for Flu B detection, enabling all reactions to occur in a single tube without uncapping, thereby eliminating aerosol contamination. This system also reduced costs by 75% compared to the two-step system, primarily by halving the reaction volume (from 20 µL to 10 µL) and reducing the Cas12a concentration from 250 nM to 125 nM, which also helps suppress cis-cleavage activity of Cas12a. These improvements are critical for reducing medical costs and facilitating clinical adoption.

While PCR remains the gold standard for nucleic acid detection, high Ct values often result in ambiguous results, termed as the “gray zones” issue (32, 33). In contrast, our one-pot one-step detection systems demonstrated high specificity (98.82% and 100%, respectively) and consistencies (96.36% and 93.69%, respectively) in clinical samples, effectively avoiding the “gray zones” problem. Thus, our one-step CRISPR-based Flu B detection system is reliable and specific, with an LOD of 58 copies per test. However, the sensitivity of the one-step Flu B detection system in human clinical samples (56.25%) remains suboptimal compared to the one-pot system (81.25%). Indeed, Zhang has reported a CRISPR-LAMP platform for Flu B detection, achieving an LOD of one copy per microliter of template (34). The lower sensitivity of our system may stem from the single reaction condition and the variability in clinical samples, particularly those with low viral loads. It should not be neglected that the number of human clinical samples used in this study was relatively limited. We plan to validate the one-step Flu B detection system in a larger cohort of clinical samples in future research.

Additionally, the cis-cleavage activity of Cas12a poses a technical challenge for achieving high sensitivity in one-step reactions. In this study, we optimized the reaction temperature and the concentration of Cas12a to minimize cis-cleavage effects, thus improving the detection sensitivity on target sequences. Fortunately, other advancements are ongoing, such as the temporarily inactivated crRNA in the photocontrolled crRNA activation systems, which offer promising solutions to enhance sensitivity and specificity (35). Besides, a conjugation-free, effective, and universal detection platform was developed by pre-encapsulating various protein indicators into hydrogels, and it was capable of detecting as few as two copies per microliter genetic sequences of Flu B (36). In these settings, the RPA assay is not affected by the cleavage activity of Cas12a. With more technical advances, the one-step RT-RPA-CRISPR/Cas12a system may perform better in detecting Flu B and other RNA viruses.

In conclusion, we developed a one-pot, one-step Flu B detection system by combining RT-RPA and CRISPR/Cas12a systems in a single tube. This system operates at a constant temperature and eliminates the need for complex procedures, offering simple, rapid, accurate, and contamination-free advantages for Flu B diagnosis. Additionally, all regents can be pre-prepared via lyophilization, providing a solid foundation for developing the point-of-care diagnostic for Flu B. These findings provoke valuable insights into the applications of one-step CRISPR/Cas system in molecular diagnostics for detecting other pathogens.
